# Magnetoencephalography in the Detection and Characterization of Brain Abnormalities Associated with Traumatic Brain Injury: A Comprehensive Review

**DOI:** 10.3390/medsci9010007

**Published:** 2021-02-04

**Authors:** Geoffrey W. Peitz, Elisabeth A. Wilde, Ramesh Grandhi

**Affiliations:** 1 Department of Neurosurgery, University of Texas Health San Antonio, San Antonio, TX 78229, USA; Peitz@uthscsa.edu; 2 Department of Neurology, University of Utah, Salt Lake City, UT 84132, USA; elisabeth.wilde@hsc.utah.edu; 3 Department of Neurosurgery, Clinical Neurosciences Center, University of Utah, Salt Lake City, UT 84132, USA

**Keywords:** magnetoencephalography, traumatic brain injury, concussion, functional neuroimaging

## Abstract

Magnetoencephalography (MEG) is a functional brain imaging technique with high temporal resolution compared with techniques that rely on metabolic coupling. MEG has an important role in traumatic brain injury (TBI) research, especially in mild TBI, which may not have detectable features in conventional, anatomical imaging techniques. This review addresses the original research articles to date that have reported on the use of MEG in TBI. Specifically, the included studies have demonstrated the utility of MEG in the detection of TBI, characterization of brain connectivity abnormalities associated with TBI, correlation of brain signals with post-concussive symptoms, differentiation of TBI from post-traumatic stress disorder, and monitoring the response to TBI treatments. Although presently the utility of MEG is mostly limited to research in TBI, a clinical role for MEG in TBI may become evident with further investigation.

## 1. Introduction

Traumatic brain injury (TBI) occurs an estimated 27 million to 69 million times per year throughout the world [1,2]. The plurality of TBIs occur from unintentional falls, and most patients with TBI do not require hospitalization [3]. However, even mild TBI may result in persistent symptoms such as headache, cervicalgia, vertigo, and cognitive impairment, and the impairment may be more pronounced with repeated TBI. Furthermore, mild and sometimes moderate TBI is often undetectable via conventional imaging such as computerized tomography (CT) and anatomical magnetic resonance imaging (MRI). More advanced imaging techniques are required for diagnosis, monitoring, and research of mild TBI.

Functional imaging techniques can be useful for diagnosis and prognosis in mild TBI and for monitoring the effects of therapeutic interventions [4,5]. Functional imaging has been shown to detect mild TBI that is undetectable in conventional anatomic imaging, and long-term functional imaging abnormalities have been demonstrated in patients with chronic mild TBI [6,7,8,9]. Functional imaging has also been used to analyze patterns of disrupted connectivity and the response to therapeutic interventions in severe TBI [10,11].

Most functional imaging techniques measure neuronal activity indirectly through neurovascular or metabolic coupling, that is, to provide energy for increased neuronal activity, local cerebral blood flow, and glucose uptake increase. Neurovascular coupling is the basis for functional MRI (fMRI) with blood-oxygen-level-dependent (BOLD) imaging or arterial spin labeling (ASL) MRI. Neurovascular coupling is also the basis for single-photon emission computed tomography (SPECT), which measures gamma emissions from Technetium tc 99 m exametazime. Finally, neurovascular coupling is the basis for functional near infrared spectroscopy (fNIRS), which compares light absorbance in the wavelengths absorbed by oxyhemoglobin with the wavelengths absorbed by deoxyhemoglobin. Alternatively, metabolism coupling to neuronal activity is the basis for positron emission tomography (PET), which detects emissions from a radiotracer linked to a biologically active molecule such as fluorodeoxyglucose (FDG). Byrnes et al. have comprehensively reviewed the use of FDG-PET in TBI [12]. Although these techniques, especially fMRI, provide good spatial resolution, there is a delay between neuronal activation and the associated increase in blood flow and energy supply. Diagnostic techniques that directly measure neuronal activity, including electroencephalography (EEG) and magnetoencephalography (MEG), have superior temporal resolution.

**Figure 1:**
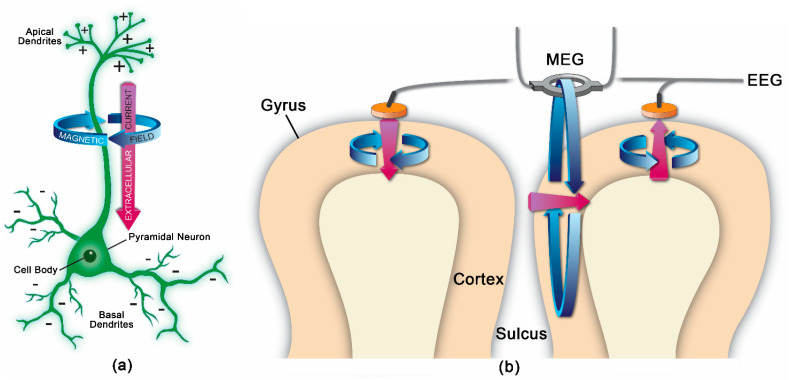
(**a**) Pyramidal neuron with positive extracellular charge around the apical dendrites and negative extracellular charge around the somatic dendrites, as in the case of a net excitatory post-synaptic potential at the somatic dendrites and/or a net inhibitory post-synaptic potential at the apical dendrites. Purple arrow represents the extracellular electric current, and blue arrows represent the magnetic field associated with the extracellular current; (**b**) Cortical gyri and sulcus with pink arrows representing electric dipoles from synchronized neuronal activity and blue arrows showing the magnetic fields associated with these electric dipoles. The MEG magnetometer only detects magnetic fields from dipoles oriented tangential to the cortical surface, whereas the EEG electrodes can detect radial and tangential electric dipoles. MEG = magnetoencephalography; EEG = electroencephalography.

**Figure 2:**
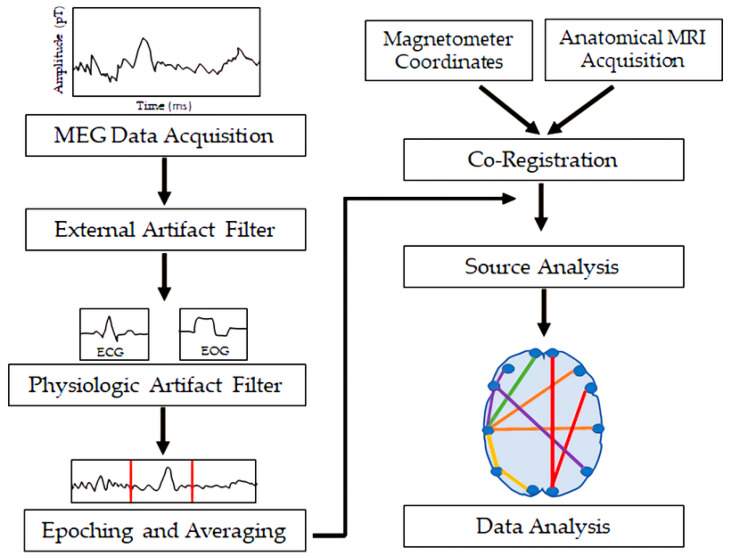
Overview of the processing steps involved in magnetoencephalography from data acquisition to data analysis. MEG = magnetoencephalography; ECG = electrocardiogram; EOG = electrooculogram; MRI = magnetic resonance imaging.

MEG measures magnetic flux on the surface of the head associated with underlying neuronal electrical currents. Magnetic signals from the brain were first recorded by the physicist David Cohen in 1968 [13]. Since then, MEG has developed into a sophisticated technique involving approximately 300 sensors on the scalp, along with appropriate shielding to minimize noise from background magnetic fields. Ion currents from post-synaptic potentials make the biggest contribution to the MEG signal; the currents include intracellular currents along the soma–dendritic axis and an opposite-direction extracellular return current [14]. To generate a magnetic field detectable by scalp sensors, simultaneous currents must occur in neurons with similar orientations. Therefore, the neocortical pyramidal neurons aligned perpendicular to the cortical surface generate the primary signals detected by MEG, but action potentials and fast sodium ion spikes may also contribute if synchronized [14]. Like EEG, MEG allows for analysis of neural oscillatory activity across a range of frequency bands (e.g., delta, <4 Hz; theta, 4–8 Hz; alpha, 8–12 Hz; beta, 12–30 Hz; gamma, >30 Hz). MEG has a similar temporal resolution to EEG, but MEG is less susceptible to distortion from variations in tissue conductivity than EEG [14]. EEG, however, can detect both radial and tangential currents, whereas MEG only detects the tangential component of currents. Therefore, MEG and EEG may be combined for optimal neuronal signal detection. Figure 1 demonstrates the orientation of electrical currents and magnetic fields in relation to MEG and EEG sensors. Figure 2 demonstrates an overview of the MEG processing steps, including filtering external and physiological artifacts, identifying epochs and averaging signal from like epochs, and coregistration of the MEG sensor coordinates with anatomical MRI for source analysis.

Given the high incidence and prevalence of TBI and the need for new technologies for the diagnosis, prognosis, and monitoring response to treatments in TBI, this review examines the current evidence for the use of MEG in TBI.

## 2. Literature Search

The authors conducted a PubMed search using the Medical Subject Headings (MeSH) “traumatic brain injury” and “magnetoencephalography” and the filter for human studies. The articles were screened by reviewing their abstracts for relevance to clinical and research applications of MEG in adults with TBI. Articles were included in the review only if the full text was available in English.

There were 22 articles in PubMed fitting the search criteria. Screening the abstracts yielded 18 articles relevant to clinical and research applications of MEG in people with TBI. One of these articles was excluded because the full text was not available in English. From the reference lists of the articles found in the PubMed search, we identified 11 additional original research articles on the use of MEG in TBI. Together, the articles described the use of MEG for detecting TBI, differentiating TBI from conditions with similar features, characterizing changes in brain rhythm or connectivity from TBI, correlating imaging findings with clinical features, and monitoring the response to treatments. These applications are described in detail herein. Participant demographics for each study are shown in Table 1, and MEG system and data analysis details are available in Table 2.

## 3. Detection of TBI

Whereas most patients with severe TBI have hemorrhage, edema, and/or ischemia detectable via CT or anatomical MRI, many mild and sometimes moderate injuries do not have features visible with these conventional techniques. As with other functional imaging techniques, MEG has been shown to detect TBI that is otherwise silent in conventional imaging. MEG features indicating mild TBI include abnormal resting state neural oscillations and altered connectivity between brain regions, as described in detail in this section.

Among the first to investigate MEG in TBI, Lewine et al. in 1999 compared conventional MRI, EEG, and resting-state MEG findings in 20 normal participants, 20 symptomatic post-concussion patients, and 10 asymptomatic post-concussion patients [15]. The post-concussion patients’ injuries occurred 2–16 months prior to the study. Compared with MRI and EEG, MEG was significantly more sensitive for abnormalities in post-concussive patients, and all of the patients with MRI abnormalities also had MEG abnormalities. The MEG abnormalities were mostly characterized by abnormal low-frequency magnetic activity (ALFMA). Similarly, Lewine et al. (2007) retrospectively reviewed MRI, SPECT, resting-state MEG, and neuropsychological testing findings in 30 patients with symptoms at least one year after mild blunt head trauma [16]. MEG was significantly more sensitive than MRI or SPECT for identification of abnormalities in patients with cognitive symptoms, and the notable MEG feature in this study was abnormal dipole slow-wave activity. Furthermore, the functional brain region in which the abnormalities occurred in each patient correlated with the patient’s specific cognitive symptoms (e.g., frontal lobe abnormal dipole slow-wave activity in patients with executive dysfunction). These studies set the stage for MEG as an important tool in the diagnosis of mild TBI and in highlighting the anatomic correlation among patients with even mild functional deficits.

Further studies have confirmed that resting-state MEG is sensitive for detecting mild TBI. Huang et al. (2009) analyzed MEG integration with diffusion tensor imaging (DTI) for detection of mild TBI in a case–control series of 10 patients with subacute to chronic mild TBI and persistent post-concussive symptoms for an average of 17 months and 14 matched controls [17]. Abnormal MEG slow waves were found in all 10 patients with mild TBI, whereas only seven patients with mild TBI had abnormalities in DTI and only one patient with mild TBI had abnormal conventional MRI findings. Furthermore, the grey matter surface area generating MEG slow waves was strongly correlated with the volume of non-major white matter tracts with reduced DTI anisotropy; major white matter tract injury was associated with much higher grey matter surface area with slow waves. The authors theorized that pathologic slow waves were a result of grey matter deafferentation from white matter tract injury, as has been shown in animal studies with EEG [43,44]. Later, in 2012, Huang et al. reported results from an automated abnormal low-frequency magnetic activity (ALFMA) detection method in 45 patients with mild TBI and 10 patients with moderate TBI [18]. Time from injury to the study ranged from 4 weeks to 3 years. ALFMA was detected in 87% of patients with mild TBI and 100% of patients with moderate TBI, and the number of cortical regions with ALFMA was significantly correlated with total post-concussive symptom scores. Later, Huang et al. (2014) developed voxel-based whole-brain MEG slow-wave imaging and used it to compare 84 patients with persistent symptoms from subacute to chronic mild TBI (ranging between 4 weeks to 5 years post-injury) with 79 controls [19]. They reported an 84.5% detection rate for mild TBI (combining blast and non-blast injury mechanisms). Again, they found a correlation between the brain region with abnormal signal and the symptoms; MEG slow waves in prefrontal areas correlated with personality change, difficulty concentrating, affect lability, and depression.

Beyond comparing MEG rhythm abnormalities among specific brain regions, the connectivity between brain regions has also been used to detect TBI. Studies by Zouridakis et al. and Vakorin et al. used resting-state MEG network connectivity and machine learning to detect mild TBI [20,21]. Patients in the Zouridakis et al. study sustained TBI more than 3 months prior to the study, whereas patients in the Vakorin et al. study sustained TBI less than 3 months prior to the study. Both groups of researchers recorded resting-state MEG activity in patients with mild TBI and age- and sex-matched controls. Zouridakis et al. used Granger causality to assess connectivity between brain regions, whereas Vakorin et al. used phase locking values (PLVs, values between 0 and 1, representing the degree of phase synchrony between two brain regions) to assess connectivity. Both groups used support vector machines to classify patients as having had a mild TBI or not. The method used by Zouridakis et al. detected mild TBI with 85% accuracy. The method used by Vakorin et al. detected mild TBI with 88% accuracy, and the classification confidence was correlated with TBI symptom severity scores. A different study published in 2015 by Dimitriadis et al. also used machine learning with PLVs from resting-state MEG data to classify patients with acute, mild, or no TBI [22]. Instead of a support vector machine, they used the extreme learning machine classifier, and they reported 100% classification accuracy. The findings of these investigators demonstrated that combining MEG connectivity analysis with machine learning can represent a powerful tool for detecting mild TBI.

In another study of brain region connectivity with resting-state MEG, Kaltiainen et al. included data from the same participants at multiple points in time. The authors analyzed resting-state MEG in 26 patients with mild TBI that occurred 6 days to 6 months prior to the study [23]. In 12 of the patients, follow-up MEG was done 6 months later. Of the 26 patients, seven had abnormal low-frequency MEG activity greater than 2 standard deviations from the mean of that seen in 139 controls. At 6-month follow-up, however, only three of those seven patients had persistently abnormal low-frequency MEG activity. Li et al. (2018) similarly reported attenuation of MEG abnormalities over time in patients with mild TBI [24]. Using resting-state MEG signal source analysis and Granger causality to determine in-going and out-going connections between brain regions, they compared connectivity networks between 13 patients with mild TBI and eight matched controls. There were significantly more strong connections in the delta frequency band in patients with mild TBI, but the difference dissipated over three successive visits for MEG recording. These studies suggest a temporal relationship for the presence of abnormal MEG signals and indicate a potential for MEG to be used for mild TBI diagnosis and also as a radiological biomarker for brain “healing.” This will be discussed further in the “Monitoring Response to Treatments” section of this review.

Beyond resting-state MEG, task-based MEG has also proven useful in detecting TBI. Tormenti et al. published data from MEG recordings during a language and spatial task in five patients with a history of concussion within the past 4 months and five controls [25]. The participants responded with whether each of a series of three figures containing geometric shapes matched the preceding sentence (e.g., “The blue square is below”). They pressed a button after each word in the sentences and for each response on figures matching the sentences or not. The researchers recorded button response times and MEG activity from about 100 brain recording sites per participant. They used statistical analyses to construct classification rules to categorize patients as concussed or not based on MEG data. Compared with controls, patients with previous concussions had significantly different normalized response times to the words and figures presented in the trials. Pairing MEG data from the occipitoparietal and temporal regions, the researchers constructed a rule that correctly categorized eight of the 10 patients as concussed or not.

Da Costa et al. (2015) also used task-based MEG in 16 patients with mild TBI within 2 months of the study [26]. Patients with mild TBI showed delayed reaction times and different sequences of brain region activation compared with matched controls performing the same tasks.

In conclusion, both resting-state and task-based MEG have proven sensitive for detecting TBI even when conventional CT or MRI is normal. MEG may even be more sensitive than other advanced imaging modalities such as SPECT. In the resting state, abnormal low-frequency MEG signals are associated with TBI, and the brain regions with the low-frequency signals correlate with the injury locations and symptoms. Moreover, MEG may be used as an adjunctive study for determining recovery from brain injury.

## 4. Differentiating Mild TBI from Post-traumatic Stress Disorder

Whereas abnormal low-frequency MEG activity may be sensitive for detecting TBI, it is not specific to TBI. Other neurologic and psychiatric conditions also feature abnormal low-frequency activity, including brain tumors, infarcts, epilepsy, Alzheimer disease, and schizophrenia [45]. Clinical history can diagnose epilepsy, and anatomical imaging such as CT and MRI can be used to diagnose brain tumors and infarcts. However, dementia and psychiatric disorders cannot always be differentiated from mild TBI using conventional diagnostic methods.

Contrasting brain abnormalities between post-traumatic stress disorder (PTSD) and TBI is especially relevant because these conditions are frequently comorbid, and mild TBI may exacerbate PTSD symptoms [46,47,48]. Postconcussive symptoms, including anxiety or depression, apathy, changes in personality, dizziness, fatigue, headaches, irritability, and sleep disturbance, are commonly attributed to TBI, but the symptoms overlap with those of PTSD [49]. Clinically, it is important to distinguish between the two diagnoses because the natural history differs between psychological conditions and neurostructural damage. Therefore, attributing PTSD symptoms to TBI could give patients and providers the wrong expectations for recovery [49]. Furthermore, the treatments for each condition are different. Dunkley (2015) theorized that the two disorders could be distinguished based on MEG and that increased high-frequency phase synchronization seen in PTSD could be the result of a psychological state, whereas increased low-frequency amplitude coupling in mild TBI could be the result of neurostructural alteration [50].

Building on this theory, Popescu et al. published a study in 2016 in which they analyzed resting-state MEG recordings in 32 active-duty service-member patients with mild TBI and persistent post-concussive symptoms [27]. Each patient completed the PTSD Check List—Military version (PCL-M); 15 had low PTSD symptoms severity (PCL-M scores ≤44) and 17 had high PTSD symptom severity (PCL-M scores >44). To reduce the effect of confounding psychological conditions, the researchers excluded patients with depression and matched patients in the high and low PTSD symptoms severity groups for scores of generalized anxiety. They found reduced MEG activity in the alpha frequency band in the dorsolateral prefrontal cortex of patients with high PTSD symptom severity compared with those with low PTSD symptom severity. The study showed that reduced alpha activity in the prefrontal cortex may be a biomarker for PTSD in patients with mild TBI, but it was not designed to elucidate the significance of the prefrontal alpha activity in the pathophysiology of PTSD in patients with TBI.

In another study investigating MEG activity in veterans with mild TBI and PTSD, Rowland et al. examined six veteran patients with both PTSD and chronic mild TBI, six patients with only chronic mild TBI, six patients with only PTSD, and 10 controls [28]. The participants completed the PCL-M. The researchers recorded resting-state MEG signals, then identified nodes of peak activity in the brain. They measured phase consistency between each pair of nodes to create functional connectivity networks for each participant. Analyzing these networks, they found that patients with PTSD had decreased structure and increased randomness in their functional connectivity networks. Patients with mild TBI had greater structure and less randomness. The authors’ methods could be useful in differentiating mild TBI and PTSD, which are often comorbid in military patient groups as well as intimate partner violence patient groups. However, as described in the Fifth Independent Medical Expert Group report, further research is required before MEG can be used as a diagnostic test for TBI or PTSD [51].

In a step closer to using MEG clinically to diagnose PTSD, Zhang et al. (2020) developed a machine learning classifier to identify military service members with PTSD [29]. They compared resting-state MEG data from 23 soldiers diagnosed with PTSD with those from 21 soldiers without PTSD but who had similar traumatic experiences in battlefield deployment. The support vector machine (SVM) area under the receiver operating characteristic curve was 0.9, demonstrating very good accuracy. However, this study did not directly look for TBI in conjunction with or as opposed to PTSD, so additional research is still needed for MEG to distinguish TBI from PTSD accurately.

## 5. Characterizing Connectivity Abnormalities and Correlating with Clinical Features

Although detecting mild TBI and differentiating it from other conditions with similar features can be clinically important for tailoring existing treatment regimens, understanding the pathophysiology of persistent symptoms after TBI is important to developing and testing new treatment modalities. Previous studies have used both resting-state and task-based EEG to study the effect of TBI on brain rhythm and connectivity, and MEG has also become useful for this [52,53,54,55]. As discussed in the previous sections, researchers have used MEG connectivity analyses with a focus on detecting and differentiating TBI [17,20,21,22,23,24,25,26,27,28,50]. In this section, we will focus on discussing the use of MEG connectivity analysis studies in exploring the pathophysiology of persistent symptoms after TBI.

Luo et al. (2013) were among the first to show a correlation between MEG signal and specific cognitive symptoms in patients with TBI [30]. Using resting-state MEG data from 18 military veterans with known or suspected TBI at least 6 months prior to the study and 18 age- and sex-matched controls, they applied Lempel–Ziv complexity (LZC), which estimates complexity by the number of patterns in finite sequences. Participants were also administered a number of neuropsychological tests, producing 44 neuropschological values representing attention, executive function, global cognitive function, language, memory, motor functions, processing speed, and visual–spatial skills. Not only were there multiple brain regions with significantly lower LZC in patients with TBI compared with controls, but there were also four neuropsychological values that significantly correlated with LZC in distinct brain regions. The functional significance of positive vs. negative correlations between LZC and neuropsychological values requires further investigation, but the results from this study suggest that LZC applied to MEG may be a tool for analyzing motor, reasoning, and visual perception dysfunction after TBI.

As described in the section on TBI detection, Dimitriadis et al. (2015) analyzed MEG connectivity based on PLVs with machine learning to detect mild TBI with high accuracy, but they also used graph theory to better characterize the different regional brain network connectivity between patients with mild TBI and age-matched controls [22]. The control participants had strong local connections and some long-range connections that accounted for 20% of the total number of identified connections. Conversely, patients with mild TBI had weak local connections but strong long-range connections that accounted for 60% of the total. Furthermore, the long-range connections in the controls mainly linked frontal to central regions or central to peripheral regions, but the long-range connections in patients with mild TBI mainly linked peripheral regions. Dunkley et al. (2015) and Antonakakis et al. (2016, 2017) also showed differences in brain connectivity across multiple frequency bands between patients with mild TBI and controls [31,32,33].

In 2020, Antonakakis et al. studied spontaneous network microstates in patients with TBI [34]. Using resting-state MEG data from 30 patients with mild TBI and 50 healthy controls, they constructed dynamic functional connectivity graphs from PLVs. Subsequently, they used a vector quantization process to compute network microstates for each participant and analyzed how these network microstates changed over time segments. Then, using a machine learning classifier with network microstate features, they reported 94% accuracy in classifying patients with mild TBI and healthy controls. The patients with mild TBI had lower time-resolved organization in their brain connectivity networks compared with controls.

A study by Alhourani et al. (2017) also used PLVs from resting-state MEG data to characterize functional connectivity in patients with chronic, mild TBI (3–96 months after injury) and persistent post-concussive symptoms [35]. Using graph theory, they found that local communication efficiency was reduced in all frequency bands in patients with mild TBI compared with controls. The researchers also reported reduced connectivity predominantly in the parietal and occipital lobes of patients with mild TBI compared with controls. They noted that specific regions with significantly reduced connectivity included major hubs in the default mode network (DMN), a network active during rest and associated with memory and attention [35]. In 2018, Dunkley et al. expanded on the significance of the DMN in TBI [36]. The researchers obtained resting-state MEG recordings in 20 patients with a concussion within 3 months prior to the study and 20 age- and sex-matched controls. The participants also underwent cognitive behavioral testing including the Sports Concussion Assessment Tool 2 (SCAT-2). The researchers calculated amplitude envelope correlations (AECs), which are values measuring the temporal correlation between waveform amplitudes in separate brain regions, independent of their phases. They used the AECs to construct graphs of intrinsic connectivity networks. As opposed to Alhourani et al. (2017), they found increased DMN connectivity in the alpha and beta frequency bands in patients with previous concussions. Furthermore, after controlling for comorbidities, the authors found that DMN connectivity had a significant, positive correlation with concussion symptoms.

Another study correlating post-concussion symptoms with brain connectivity findings was published by Popescu et al. in 2017 [37]. Rather than using a general symptom scale score, these authors focused on word finding difficulty associated with mild TBI. They recorded task-based MEG data in 57 right-handed military service members with a history of mild TBI and persistent symptoms who were stratified into three cognitive performance groups based on the Rivermead Behavioral Memory Test. During MEG recording, the participants were presented with 80 pictures of common objects and tasked with naming them. The authors reported early activation of widely distributed networks for visual and linguistic processing in the dominant hemisphere after presentation with an object to be named. They also found widespread decreased amplitude of the response in patients with mild TBI and low cognitive performance scores compared with those with mild TBI and medium or high cognitive performance scores.

Finally, MEG can also be combined with EEG to characterize activity in the brain associated with TBI. In 2015, Li et al. published a study in which they analyzed resting-state MEG and EEG activity at 68 brain regions of interest [38]. Comparing brain activation maps between patients with mild TBI and controls, they found significant differences in low-frequency activity on both EEG and MEG. The sample size was limited to six patients with mild TBI and five controls, but further research combining EEG and MEG may prove useful in characterizing detailed rhythm and connectivity abnormalities after TBI and explaining how these abnormalities relate to symptoms.

## 6. Monitoring Response to Treatments

Multiple studies have shown that MEG abnormalities in TBI dissipate with time since the injury and/or with recovery from the injury [23,24,56], and others have demonstrated that MEG abnormalities correlate with symptoms severity [18,21]. Further studies have taken an additional step by incorporating treatment programs into the research protocol and using MEG to look for responses to the treatments. This is an important area of research that could have significant clinical implications.

In early studies on response to TBI treatment, Castellanos et al. (2010) compared resting-state MEG recordings in patients with chronic TBI (range 2–6 months after injury) before and after a neuropsychological rehabilitation program [39,40]. The patients had severe cognitive impairment, and the rehabilitation programs involved cognitive therapy and lasted 7–12 months. The authors used graph theory to analyze MEG data and found that, compared with controls, patients with TBI had increased network strength in the delta frequency band and decreased network strength plus network reorganization in the alpha frequency band [39]. After rehabilitation, the network characteristics in each frequency band became more similar to those of the control participants. In 2011, Castellanos et al. reported similar network changes in patients pre- and post-rehabilitation for chronic TBI (range 4–6 months after injury) but also found correlations between region-specific connectivity values and neuropsychological test scores for various cognitive functions. These studies identified parameters that are correlated with pre- and post-rehabilitation status in TBI patients, but they were not designed to determine whether time since injury or the rehabilitation program was responsible for the MEG changes and cognitive recovery.

MEG has also been used to evaluate a more specific therapeutic intervention, perception attention therapy (PATH), which is a visual timing training task involving movement discrimination. Lawton and Huang (2019) studied four patients with cognitive deficits attributed to TBI who underwent working memory task-based MEG before and after 8 weeks of PATH neurotraining [41]. After the intervention, the MEG activity during the working memory task increased in the participants’ frontoparietal attention networks and dorsal stream, as did their performance on standardized tests of attention, reading, and working memory skills.

Besides rehabilitation programs requiring active participation, a passive TBI treatment intervention has also been tested with MEG. Huang et al. (2017) recorded MEG data in patients with chronic TBI and persistent post-concussive symptoms (average duration 48 ± 25 months) before and after a 6-week passive neurofeedback intervention using low-intensity pulse transcranial electrical stimulation with EEG monitoring [42]. They reported reductions in both abnormal MEG slow waves and post-concussive symptom scores after the intervention. Similarly to the studies by Castellanos et al., this study lacked a null/sham intervention group so whether the intervention or the timing was responsible for the changes is unclear. However, both research groups have shown that MEG can be used as a supplement to neuropsychological testing to assess recovery after TBI and that MEG parameters could be used as outcome measures for future studies assessing the efficacy of TBI clinical treatment interventions.

## 7. Limitations of MEG

Although the temporal resolution of MEG is very good, its spatial resolution is inferior to that of MRI. For functional brain activity maps, MEG data are often co-registered with anatomical MRI for MEG source imaging, requiring patients/participants to undergo an MRI scan in addition to the MEG data acquisition session. However, even with MRI-aided source modelling, Kaltiainen et al. (2018) demonstrated limited sensitivity of MEG to traumatic lesions deep in the brain (>3 cm from the cortex) [23]. Therefore, functional imaging methods with strong spatial resolution such as fMRI are important complements to MEG in TBI research. Additionally, longitudinal studies that assess the progression of MEG symptom abnormalities and the relation of these signals to cognitive and other outcome over time are quite limited; this was identified as a major gap in the clinical utility of MEG in TBI in a consensus panel summit convened by the United Kingdom Office of the Surgeon General in 2020 to specifically address the relative utility of MEG and other imaging techniques [57].

MEG machines are also very expensive and require a magnetically shielded room. Specialized expertise is also required for analysis and interpretation. Thus, it is not available in many institutions. The benefit of MEG as an adjunct in choosing depth electrode sites for epilepsy surgery planning may be worth the additional cost [58]. However, MEG does not yet have a concrete clinical application in TBI with a cost–benefit ratio proven favorable.

## 8. Conclusions

MEG is a functional brain imaging technique with high temporal resolution and reasonable spatial resolution when co-registered with anatomical MRI. TBI researchers have demonstrated a variety of uses for MEG, especially in the detection of mild TBI and the characterization of functional network connectivity changes from TBI that cannot be seen with conventional anatomical imaging techniques. Combining MEG with other techniques, such as EEG or DTI, can support efforts to understand connectivity changes from TBI. MEG research has also been directed toward differentiating TBI from other conditions with similar clinical features, such as PTSD, as well as toward assessing responses to TBI treatment interventions. Although presently the utility of MEG is mostly limited to research in TBI, future research may eventually identify a clinical role for it in TBI as it has in epilepsy.

## Figures and Tables

**Table 1:** Participant demographics for each study, listed in the order referenced. Sets are ranges unless otherwise specified with “mean.” Fields marked with a hyphen indicate the information was not available. “N/A” indicates “not applicable.” * Retrospective review. ‡ Applies to affected patient group only. ^ Indicates years with post-traumatic stress disorder symptoms as opposed to years since traumatic brain injury.

Study	No. of Affected Patients	No. of Healthy Controls	% Female	Ages	Time Since Injury
Lewine (1999) [15]	30	20	44	18–57	2–16 months
Lewine (2007) [16]	30	N/A *	-	≥18	≥1 year
Huang (2009) [17]	10	14	10 ‡	12–43	1–46 months
Huang (2012) [18]	55	44	16	Mean 27 ± 8	4 weeks–3 years
Huang (2014) [19]	84	79	16	Mean 28 ± 9	4 weeks–5 years
Zouridakis (2012) [20]	10	10	30	20–46	>3 months
Vakorin (2016) [21]	20	21	0	21–44	<3 months
Dimitriadis (2015) [22]	31	55	42 ‡	Mean 29 ± 9	<24 h
Kaltiainen (2018) [23]	26	139	68	18–60	6 days–6 months
Li (2018) [24]	13	8	48	Mean 26	-
Tormenti (2012) [25]	5	5	50	16–57	≤4 months
Da Costa (2015) [26]	16	16	0	20–40	2 months
Popescu (2016) [27]	32	N/A	0	Mean 40	6 months–11 years
Rowland (2018) [28]	18	10	0	Mean 39 ± 10	Mean 6 ± 3 years
Zhang (2020) [29]	23	21	0	18–48	1–4 years ^
Luo (2013) [30]	18	18	0	Mean 29 ± 6 ‡	≥6 months
Dunkley (2015) [31]	20	21	0	Mean 31 ± 7 ‡	<3 months
Antonakakis (2016) [32]	30	50	43 ‡	Mean 29 ± 9 ‡	<24 h
Antonakakis (2017) [33]	30	50	43 ‡	Mean 29 ± 9	<24 h
Antonakakis (2020) [34]	30	50	43 ‡	Mean 29 ± 9	<24 h
Alhourani (2017) [35]	9	15	44 ‡	14–62	3 months–8 years
Dunkley (2018) [36]	26	24	0	Mean 31 ± 7 ‡	<3 months
Popescu (2017) [37]	80	N/A	1	Mean 59	-
Li (2015) [38]	6	5	36	Mean 29 ± 7	-
Castellanos (2010) [39]	15	14	-	18–51	4–6 months
Castellanos (2011) [40]	15	14	13	18–51	2–6 months
Lawton (2019) [41]	4	N/A	0	15–68	-
Huang (2017) [42]	6	N/A	17	27–41	Mean 48 ± 25 months

**Table 2:** Magnetoencephalography system and data analysis details for each study, listed in the order referenced. MNI = Montreal Neurological Institute; SVM = support vector machine; CTF = CTF MEG International Services; ELM = extreme learning machine; ANOVA = analysis of variance; AAL = Automated Anatomical Labelling atlas; LOOCV = leave-one-out cross-validation; AUR = area under receiver operating characteristic curve; rRF = recursive random forests; k-NN = k nearest neighbors; ENS = ensemble classification; AEC = amplitude envelope correlation.

Study	MEG System	Sensor/Source Space; Atlas, if Applicable	Functional Connectivity	Data Analysis	Features Selection if Machine Learning	Classifier if Machine Learning
Lewine (1999) [15]	Magnes	Source	N/A	Z-score	N/A	N/A
Lewine (2007) [16]	Elekta	Source	N/A	Fisher exact test	N/A	N/A
Huang (2009) [17]	Elekta	Source	N/A	Nonparametric permutation tests	N/A	N/A
Huang (2012) [18]	Elekta	Source; MNI-152	N/A	Correlation coefficient	N/A	N/A
Huang (2014) [19]	Elekta	Source; MNI-152	N/A	Z-score	N/A	N/A
Zouridakis (2012) [20]	Magnes	Sensor	Static	Machine learning	Fisher’s criterion ranking	SVM
Vakorin (2016) [21]	CTF	Source; AAL	Dynamic	Machine learning	LOOCV	SVM
Dimitriadis (2015) [22]	Magnes, Elekta	Sensor	Static	Machine learning	Tensor space dimensionality reduction	ELM
Kaltiainen (2018) [23]	Elekta	Source	N/A	Chi square	N/A	N/A
Li (2018) [24]	CTF	Source	Static	ANOVA	N/A	N/A
Tormenti (2012) [25]	Elekta	Source	N/A	Task-based activity, stepwise linear discriminant analysis	N/A	N/A
Da Costa (2015) [26]	CTF	Source	N/A	Task-based activity, *t*-test, ANOVA	N/A	N/A
Popescu (2016) [27]	Elekta	Source; Desikan-Killiany	N/A	*t*-test, Mann–Whitney rank-sum, Spearman’s rank correlation coefficient	N/A	N/A
Rowland (2018) [28]	CTF	Source	Static	Graph theory metrics, ANOVA	N/A	N/A
Zhang (2020) [29]	CTF	Source; AAL	Static	Machine learning, AUR	rRF	SVM
Luo (2013) [30]	Magnes	Sensor	N/A	Lempel-Ziv complexity, *t*-test	N/A	N/A
Dunkley (2015) [31]	CTF	Source; AAL	Static	AEC, nonparametric permutation tests	N/A	N/A
Antonakakis (2016) [32]	Magnes	Sensor	Static	Machine learning	Tensor subspace analysis	k-NN, ENS, ELM
Antonakakis (2017) [33]	Magnes	Sensor	Static	Machine learning	Iterative bootstrap	k-NN, SVM
Antonakakis (2020) [34]	Magnes	Source; AAL	Dynamic	Machine learning	Rank-feature	k-NN
Alhourani (2017) [35]	Elekta	Source; MNI-152	Static	Phase synchrony, graph theory metrics	N/A	N/A
Dunkley (2018) [36]	CTF	Source	Static and dynamic	AEC, nonparametric permutation tests	N/A	N/A
Popescu (2017) [37]	Elekta	Source; Desikan-Killiany	N/A	Normalized evoked response power, ANOVA	N/A	N/A
Li (2015) [38]	CTF	Source; Desikan-Killiany	N/A	Z-score maps	N/A	N/A
Castellanos (2010) [39]	Magnes	Sensor	Dynamic	Distance-to-control connectivity patterns, Kruskal–Wallis	N/A	N/A
Castellanos (2011) [40]	Magnes	Sensor	Dynamic	Graph theory metrics, Kruskal–Wallis	N/A	N/A
Lawton (2019) [41]	Elekta	Source; MNI-152	N/A	Task-based activity, *t*-test		
Huang (2017) [42]	Elekta	Sensor	N/A	Z-score maps	N/A	N/A

## Data Availability

No new data were created or analyzed in this study. Data sharing is not applicable to this article.
